# Beyond the ‘transition’ frameworks: the cross-continuum of health, disease and mortality framework

**DOI:** 10.3402/gha.v7.24804

**Published:** 2014-05-15

**Authors:** Barthélémy Kuate Defo

**Affiliations:** Public Health Research Institute and Department of Demography, University of Montreal, Montreal, Quebec, Canada

The planning, development and sustainable implementation of health policies and health systems ought to be based on precise measurements and understandings of prevalence and incidence of communicable and non-communicable diseases, accidents and other disabilities, given past and current demographic and epidemiological profiles in societies as well as how they are predicted to change over time. Equally crucial is the need to understand and appreciate the underling mechanisms and influential factors of these changes, and their monetary and non-monetary costs and implications to individuals, families, communities and governments in the global context. In specific contexts, it may be the interactions between factors from different levels and categories of determinants, and their timing and sequencing during the life courses, which are critical to the health of individuals and populations and how the health care system responds to health problems. To advance knowledge and promote action, various theoretical perspectives, notably the epidemiological transition theory (1, 2), have been used in an attempt to both describe and understand local, national and global patterns in demographic and epidemiological profiles within and across societies, given the multiple domains of health (3).

The epidemiological transition theory was first formulated by Abdel R. Omran (1) to describe quite accurately the shift in demographic and disease profiles reflecting historical experiences of populations in Europe and North America from the mid-18th century through the 1950s. Over the past four decades, the focus in numerous academic and research settings has been on this theoretical perspective for training and research. It has also been used extensively for research and discussion on the changing demographic and epidemiological profiles in developing countries. By and large, it has been used as a main conceptual framework in discussing how disease patterns change over time from predominantly infectious diseases to chronic non-communicable diseases. Since the 1980s, this theory has been challenged on its applicability in low- and middle-income countries (LMICs) where valid and reliable morbidity and mortality data over long time periods are often lacking or incomplete. The validity of Omran's model has also been questioned for failing to recognize and analyze the importance of cultural and social beliefs and values, political forces and health policy in understanding epidemiological profiles, especially in developing countries. Understandably, with improvements in survival at early ages in tandem with increasingly growing proportions of adult and elderly populations as well as the emergence of new infectious diseases, such as HIV/AIDS and the re-emergence of old ones such as tuberculosis, cholera, polio and dengue fever, disease and mortality patterns in populations of LMICs have been changing in unprecedented ways. These countries are largely faced with scarcity of adequate data for health policy and planning, with the double burden of communicable and non-communicable diseases, with their health systems still mainly ill-prepared to face the challenges of quality care and affordable health care services, with a sizeable proportion of their populations living in chronic poverty and unmet basic needs, with no access to clean water, and with inexistent or sub-standard sanitation systems. Over the past 20 years or so, these compounding situations have given rise to renewed interest in the patterns of demographic and epidemiological profiles of developing countries and whether existing theoretical perspectives in global health research can provide some broad guidance. In response to those concerns, the United States National Academy of Sciences organized two workshops on the epidemiological transition in developing countries. One was in 1991 and led to the publication of the workshop proceedings (4); another was convened in 2011 on the topic of epidemiological transition in sub-Saharan Africa and resulted in a workshop summary (5). These two workshops and resulting publications highlight the continued interest in the epidemiological transition as a broad research theme in global health.

This Special Issue of *Global Health Action* is timely to reassess the epidemiological transition theory coined by Omran over 40 years ago and to consider whether it still serves the purpose it was intended for. In doing so, this Special Issue contributes most directly to the fast-growing literature on global health research in the field of population health. It contains 12 articles including this editorial, which provide the latest evidence and brings forth issues related to the epidemiological transition, in response to a call for papers by *Global Health Action* for the Special Issue on ‘Epidemiological Transitions: Beyond Omran's Theory’.

This editorial first gives an overview of the other 11 articles published in the Special Issue. These articles review the original formulation of Omran's theory and its applications, reappraise its utility and relevance to contemporary developing countries, and consider its usefulness for current and future demographic and epidemiological changes. Then, with reference to developing countries and African countries more specifically, we discuss the inadequacies of theoretical perspectives – demographic transition, epidemiological transition and health transition – since these perspectives have generally been used to inform the understanding of global health researchers regarding the multilevel influences on population change and epidemiological landscape in societies over time. Next, we propose a new framework that is better suited for guiding and illuminating historical, contemporary and future demographic and epidemiological changes in human populations, especially in LMICs. Special reference is made of African countries where individuals and populations, on average, have been falling excruciatingly behind by most indicators of well-being and development. Finally, we discuss ways in which the new framework and consequent multilevel life course data collection and analyses might inform understandings on underlying mechanisms of demographic and epidemiological changes and the responses of the health system to them.

Together with the editorial, these articles cover the several broad topics related to the epidemiological transition. The first three papers clarify the concepts of the epidemiological transition in parallel with those of the demographic and health transitions, followed by reviews and empirical assessments of their relevance in Africa and other parts of the developing world. My paper starts by presenting similarities and differences between the three perspectives of the demographic transition, the epidemiological transition and the health transition (6). It then considers seven specific conjectures emanating from these perspectives and empirically tests each of them with time series data covering 60 years of population change and mortality statistics for all five regions and 57 countries of Africa, along with cause-of-death data. It is shown that existing concepts provide inadequate frameworks for describing and understanding population health trends in Africa in general and specifically in sub-Saharan African countries. With the notable exception of island African countries such as Mauritius and probably some northern African countries, the overwhelming evidence indicates that over the past 60 years, African countries have not experienced any sustained shift from one epidemiological regime to another nor seen demographic changes and health improvements as predicted from the perspectives of the demographic, epidemiological, and health transition, in contrast to prevailing situations in other developing countries outside of Africa.

In a review of published studies from 1971 to 2013 on mortality transition and associated epidemiological changes in diverse contexts of LMICs, Santosa et al. document substantial variation in empirical evidence supporting and contradicting Omran's original propositions, and underscore the critical role of social determinants of health in contributing to deviations from these propositions (7). They synthesize some new evidence of such deviations, such as in Nauru where high mortality from infectious diseases gave way to quite high mortality from diabetes, circulatory disorders and accidents over a short period without any appreciable increases in life expectancy; in Mexico with overlapping burdens of disease and an increasing trend of non-communicable diseases at younger ages during 1922–1955 due to poverty and unaffordability of healthcare; and in the native Indian population in Canada following a different epidemiological profile from the general population. They stress the need for a new evidence-based framework of patterns of changes in causes of death and disruptions in health due to emerging risks, which focuses on the underlying mechanisms and cause-specific mortality changes that result.

Zuckerman et al. argue that from anthropological and epidemiological perspectives, the original epidemiological transition theory first formulated by Omran has several limitations (8). Hence, they modify it within an expanded evolutionary framework. They label the ‘first epidemiological transition’ to coincide with the Neolithic Period and the Agricultural Revolution, the ‘second epidemiological transition’ to typify the Omran's classic formulation of the epidemiological transition theory, and the ‘third epidemiological transition’ to represent the situation of emerging and re-emerging infectious diseases occurring in the modern era. They do their categorization by using the hygiene hypothesis to explain the increased incidence of chronic inflammatory diseases (CIDs) such as allergic and autoimmune diseases or to explain the emergence and re-emergence of infectious disease rise in CIDs; the concept of a third epidemiological transition is used to explain the increase in emerging and re-emerging infections. Building from the socio-ecological model recognizing that a broad array of systems and interrelated determinants of health operate either synergistically or antagonistically in modern epidemiology, they discuss the implications of their categorization for the understanding of the complex and multiple dimensions of health and disease over time as well as for clinical practice, global health policies, and future epidemiological research which can contribute to improving population health.

Since the International Conference on Population and Development in Cairo in 1994, the agenda of sexual and reproductive health has been brought to the forefront in women's health in LMICs. Yet, with rising aging populations and higher proportions of females than males surviving to old ages, non reproductive health conditions such as non-communicable diseases are becoming a fundamental public health concern in those countries. Using the example of depression and Type 2 diabetes co-morbidity in India, Mendenhall and Weaver establish that women are increasingly confronting these diseases within the complexities of the full spectrum of health concerns covering invariably communicable and non-communicable conditions (9). They call into question the existing paradigm of diseases categorization and propose a movement away from the traditional distinctions between ‘chronic’ and ‘acute’, ‘communicable’ and ‘non-communicable’ diseases; they make the case that in fact these conditions often occur together in most societies of LMICs. They echo the call for a move beyond diseased-focused model in public health to new public health paradigms rethought in light of the challenges of aging populations. Mendenhall and Weaver argue that women living in LMICs have distinctively unique experiences as they face social and health problems compared to women living in developed nations. They warn against bias of research from high-income nations in construing LMIC women's experiences and contributing to knowledge displaced from women's social experiences or policies and programs disconnected from the social, economic, and cultural factors surrounding women's mental and physical health problems in LMICs often due to socially-driven inequalities. They argue for a life course approach encompassing the role of social and economic determinants of health in women's lifetimes. Mendenhall and Weaver advocate an integrative approach that is *health*-focused as opposed to the disease-focused approach which dominates clinics and public health agendas as well as global health dialogues and funding structures, as the co-occurrence of mental and physical health problems gains recognition in the public health agenda, a more nuanced understanding of sociocultural influences on women's lifetime health is crucial.

Two studies focus on India. Yadav and Arokiasamy assess the structural changes in the patterns of morbidity and mortality in India for understanding India's progress in epidemiological transition (10). They find structural changes in disease patterns concomitant with the transformation in the age pattern of morbidity and mortality. During the last four decades from 1970 to 2007, India moved quickly from the dominance of child and adult mortality to a progressive phase with the dominance of old age mortality. By the mid-1990s, the burden of communicable diseases increased considerably in adult and old ages. Using data from multiple sources, they suggest that all geographical regions of India have experienced a rise in morbidity accompanied with marked fall in mortality, despite notable heterogeneity among the states (e.g. highest morbidity rate of 255/1,000 persons in Kerala and lowest morbidity rate of 33/1,000 persons in poorer states of Jharkhand).

The secular decline of mortality in Kerala during the last century set this Indian state apart from the others and made Kerala a success story by most accounts. Thomas and James examine the pattern of mortality by cause of death and associated changes in human longevity in Kerala since the beginning of the 20th century, to see if changes in mortality rates and causes of death were characterized by a transition in mortality to the adult ages and if there was a shift in the patterns of causes of death from infectious to chronic, degenerative, life style diseases (11). They find that the major reduction in mortality occurred between 1951 and 1970 in Kerala. They also suggest that there is an ongoing epidemiological transition in the recent decades in Kerala whereby more deaths due to non-communicable diseases such as cardiovascular diseases, neoplasm, accidents and injuries are occurring, than from infectious diseases and maternal and child deaths.

Two studies are based on data from rural and urban settings of Africa. The relevance of the theoretical perspective of the epidemiological transition has been little assessed in urban Africa. Masquelier et al. use a rich database from monthly reports of deaths by cause (1900–1907), published estimates (1931–1951) and micro data from death registers (1976–2012) to summarize evidence on trends in mortality by cause of death in Antananarivo (Madagascar), to shed some light on the timing and pace of the mortality decline as well as on changes in cause-of-death patterns (12). They show that the onset of the secular mortality decline in Antananarivo was ascribed to anti-parasitic and anti-microbial medicine and that the health care system has played a crucial role in mortality reduction in this urban setting despite recurrent political crises and limited public resources. From a theoretical perspective, Antananarivo has experienced mortality falls, reversals and stalls over time, with important setbacks particularly in the mid-1980s and the coexistence of infectious diseases and nutritional deficiencies with non-communicable diseases. It is only after 1990 that a sustained fall in mortality from infectious diseases has been observed. Most deaths have been captured in the vital registration system of this city as far back as the 1960s in Antananarivo, and trends in under-five mortality derived from death registers tend to be consistent with estimates from Demographic and Health Surveys (DHS) for the recent periods. Hence, it is feasible to set up civil registration of death in major African cities for monitoring changes in patterns of mortality by cause and responses of the health care system performance to health problems through health interventions.

Migration and urbanization per se were not the focus in classic formulations of theoretical perspectives of the demographic transition theory or the epidemiological transition theory. With over half of the world population now residing in urban settings where natural increase is playing an increasing role in population change and distribution, the topic of migration and health in the context of the epidemiological change has gained prominence in recent years. Collison et al. take advantage of the availability of longitudinal demographic and health data on temporary rural–urban migration of rural residents from the northeast of South Africa, to analyze trends in temporary migration and mortality and how they are related over time in this setting (13). Temporary migration is related to mortality from communicable diseases, but this association is inconsistent over time. For instance, there is a strong negative association between temporary migration of males and communicable disease mortality early during the observation period; in contrast, the association of temporary migration and mortality turns positive in the latter part of the observation period. However, in this study of the evolution of the relationship between temporary migration status and causes of death where the permanent residents who formed the baseline category were not necessarily a homogenous group during the study period, several selective processes including those directly related to health could not be ruled out.

Two papers consider health policy priorities in the context of epidemiological changes. The burden of mental, neurological, and substance use disorders in South Africa like in many LMICs has been increasing over time, and co-morbidities between these disorders and other diseases including HI/AIDS, diabetes, stroke, and epilepsy make them a public health concern. Specific challenges face South Africa's mental health system and there is limited evidence on economic assessments of mental health in sub-Saharan Africa. Jack et al. summarize current understandings and highlights key knowledge gaps on the direct and indirect costs of these disorders and the cost-effectiveness of their treatment interventions, and consider how mental health services can be scaled up toward universal health coverage in South Africa (14). Their review suggests that the most cost-effective interventions incorporate mental health care into primary care or community services without the use of specialized workers. Such interventions are appealing in South Africa given the high and increasing prevalence of these disorders and comorbid chronic conditions.

Metta et al. provide a narrative review of how the existing public policy environment, health system and community actions are dealing with non-communicable diseases in Tanzania (15). Like in many African countries, there is a lack of a policy for the rising burden of non-communicable diseases within the existing health care system in Tanzania. This hampers the development and implementation of effective strategies for the prevention and control of these diseases and their risk factors at the individual, family and community levels.

Finally, one paper deals with data needs for research on the epidemiological and demographic changes. Health information is notoriously deficient in the vast majority of developing countries, notably in Africa. To reliably document and appraise epidemiological changes for suitable health policy and planning in such settings, Byass, de Savigny and Lopez propose a practical and strategic approach to health information development. This approach focusses on a minimum dataset involving three interweaved components (16). The first component entails a continuous, reliable and unbiased documentation of age- and sex-specific mortality by major causes of deaths in the population using routine civil registration with vital statistics that are enhanced with mortality surveillance systems through verbal autopsy where necessary. They provide supporting evidence of a growing capacity development for producing and using cause-of-death data at country and sub-national levels through sentinel mortality surveillance systems such as the Health and Demographic Surveillance Site (HDSS) data from the INDEPTH Network with verbal autopsy or through sample registration with verbal autopsy (SAVVY). In the absence of accurate information on cause of death, verbal autopsy methods for LMICs are becoming increasingly standardized, adapted and simplified through machine coding of causes of death. The second component is a biennial documentation of exposure to the top 10 major risk factors for the leading causes of mortality by age and sex using population-based nationally representative surveys such as the DHS being carried out since the late 1980s in these countries. The third component consists of an annual documentation of essential preventive and curative interventions for these major causes and risk factors at the district-level within national health information systems. These authors also discuss some critically important questions to be addressed, including: the cost-effective strategies for integrating these dataset within the capacity development of national health information systems; the methodological implications for upgrading national health information systems to reliably and timely capture epidemiological changes; and the ethical and political issues to ensure sustainable improvements in national health information.

Overall, this set of 11 articles provides a global and quite representative picture of a range of topics of interest to researchers, planners, policymakers and the international community. These articles shed light on trends in the changing disease and cause-of-death mortality patterns, how policymakers may use or have been using this information to make decisions about the priorities for the health sector. All these papers have emphasized the importance of collecting quality data on disease, mortality by cause of death, and risk factors that contribute to them. They have also highlighted the limitations of existing theoretical perspectives.

## Inadequacies of the demographic, epidemiological, and health transitions for global health research

Omran's concept of the epidemiological transition is situated at the confluence of the concept of the demographic transition (17) – which preceded it – and the concept of health transition (18–20) – which followed it. The common feature of the three frameworks is that mortality transition is inherent to each of them. The demographic transition embodied the mortality transition and the fertility transition, migration and other demographic phenomena being generally treated as intervening variables. The epidemiological transition is based on the mortality transition of the demographic transition and expands the scope of this transition framework by incorporating the secular changes in disease patterns in tandem with secular changes in mortality; mortality decline is expected to trigger fertility decline generally with some time lag from the onset of the mortality decline. By and large and for all useful purposes, the health transition remains an ambiguous concept, which in empirical studies has been operationalized as an extension or a revision of the epidemiological transition in low-income countries (19), middle-income countries (18), and high-income countries (20).

Notwithstanding their merits for the description of demographic and epidemiological changes and disease patterns in populations of Europe and North America through the 1950s, the critiques over the years of these frameworks in dealing with the complexity of changes in the patterns of mortality and morbidity have revealed their limitations. In essence, they are descriptive models and not explanatory frameworks, and they cannot be used either as a theory of general validity or as a technical tool for health policy and planning, especially in most environments of the developing world. This is the case especially in sub-Saharan African countries (6). One criticism of these ‘transition’ frameworks is their inflexibility in stipulating a stage-wise linear approach, treating the population as an undifferentiated unit and in an oversimplification of the transition patterns, which do not fit neatly into either historical periods or geographic locations. This stage-wise approach to the demographic transition, the epidemiology transition and the health transition, has drawn the most criticism. The linear progression they suggest is in question given a number of variants describing at different spatial scales and population sub-groups, the complexity of demographic and epidemiological changes. Another criticism concerns the timetable, thresholds and number of stages in the ‘transition’ frameworks. As we show below, there is a need for different explanatory frameworks of complex changes in health, disease and mortality for guiding the development of data collection and methods of analysis in research for action in health promotion and health policy at the global, regional, national and local levels.

### Why should the demographic transition theory be revisited?

The demographic transition is an interpretative description of historical changes in vital rates from high to low mortality and fertility and the associated trends in population growth in the process which began around 1,800 with declining mortality in Europe, in response to industrialization characterized by inherently different packages of social and economic factors. These secular changes are accepted as a definite succession of stages: during the pretransition stage, mortality and fertility are high; during the transition stage, first mortality and then fertility decline, causing a period of robust population growth followed by a deceleration to slow population growth, moving toward low fertility, long life and population aging (17, 21–23).

The demographic transition and the epidemiological transition (hence the health transition) stipulate mortality decline as a precondition for fertility decline (1, 17, 22, 24), thereby precluding the possibility that mortality declines may not be followed, with a lag of 50 years or more generally assumed, by fertility decline. But what will be the consequences if mortality declines and fertility does not? This situation has been shown to happen in several African countries and regions over the past 60 years of historically unprecedented mortality reductions throughout the continent (6). The theoretical perspectives of the demographic, epidemiological and health transitions preclude this uncovered African situation. The data from Europe also showed that once marital fertility had dropped by as little as 10%, the decline spread rapidly whether or not infant mortality had already declined (25). In fact, the demographic transition in particular has not succeeded at predicting levels of mortality or fertility or the timing of the fertility decline in Africa. This is because the initial explanation for the demographic behavior during the transition tended to be ethnocentric, relying almost exclusively on the contention that what happened to the now-developed countries should happen to other countries in some predictable fashion. This transition is expected to spread to all parts of the world with a projected completion of 2,100 (22). The influential preconditions in African countries are considerably different from what they were when the now-industrialized countries began their transition. Demographic transition theory is notoriously inappropriate for predicting or explaining past and future trends in mortality, fertility and population growth, especially in African regions and countries (6) and is pretty much of no use for population policy. Prior to undergoing the transition, few of the now-developed countries had birth rates and death rates as high as those of most African countries over the last 60 years and currently for several countries. Internal economic development emerged as a sufficient though not a necessary cause of mortality and fertility reductions in industrialized countries; in contrast, prevailing conditions of mortality declines in Africa resulted from foreign aid coupled with public health measures and medical technology brought for disease prevention and control. Moreover, the two elements which affected the onset and sustainability of fertility reduction in developing countries included government policy intervention and new levels of communication in mass media.

The second critique has to do with the culture. There are regional patterns, along cultural and linguistic lines, in fertility and mortality patterns and life expectancy trajectories among African countries. We found that these African puzzling historical patterns occur in contiguous areas and countries that are culturally similar (same language, common ethnic background, similar lifestyles), even though the levels of urbanization and economic development are different. In particular, this applies to HIV/AIDS given its regional mapping in Africa. The health, disease and mortality patterns and population change more broadly over the last 60 years in Africa occurred in the context of widely differing political, social, economic, and demographic conditions which are quite distinct from those experienced by developed countries.

Finally, the demographic transition's end-point is still far from clear and remains debatable (26). The end-point of the demographic transition was supposed to be an older stationary and stable population corresponding with replacement fertility of 2.1 children on average, zero population growth, and life expectancies higher than 70 years. The expected stabilization of population and convergence in birth and death rates has yet to emerge (26), just as the three-stage demographic transition is far from starting in many African countries (6).

### Why should the epidemiological transition theory be revisited?

The concept of the epidemiological transition was formulated as a model for integrating epidemiology with demographic changes in human populations (1, 2, 27). The sequence of events marking these changes represents an important trade-off between mortality and morbidity as a result of the interaction between epidemiological and demographic processes (28). There are several limitations to this framework.

First, for a number of scholars (18, 19, 26, 29–42), the epidemiological transition remains a conceptually weak concept in describing and explaining the epidemiology of population change. Paul Farmer has pointed out that the epidemiological transition is a deeply ambiguous framework when infectious diseases have remained so omnipresent in the global health context (43).

Second, the epidemiological transition concept is ill defined, and therefore cannot be put into operation without ambiguity, given the main problem with identifying the beginning of the epidemiological transition on the basis of changes in cause-of-death patterns (20, 44).

Third, Omran's epidemiological transition theory has been criticized for being overly focused on mortality and fertility at the expense of morbidity and its risk factors, including an insufficient account of the role of poverty in determining disease risk and mortality, especially in less developed countries (18, 20, 45, 46). This criticism is reminiscent of the Omran's concept drawing on Notestein's formulation of the demographic transition (17), just like other stage-wise formulations such as the health transition.

Fourth, there is an overemphasis on mortality rather than disease causality and morbidity, thereby failing to understand the correlation between the causes of death and the actual morbidity that people experience during their lives (35, 36). Thus, the model of epidemiological transition is compromised by the uncertain nature of the mechanisms that drive progress through the transition lives (47, 48). It has been argued that the epidemiological transition ‘fails to grasp the global nature and the historical sequence of the mortality transition as it spread’, and that it is ‘insufficiently epidemiological in that its focus was the changing causes of death rather than the changing causes of patterns of illness’ (22: 160).

Fifth, the relative role and importance of infectious diseases (IDs) and non-communicable diseases remain unsettled (43, 47–51). On the one hand, Mackenbach (36) asserts that ‘degenerative and man-made diseases’ is a misleading term for conditions such as cancer and cardiovascular diseases which have complex etiologies. On the other hand, a recent debate has emerged on the epidemiological transition regarding what should be considered as ‘infectious diseases’ (52–55). Condrau and Worboys (41: 153–154) argue that the importance of infectious diseases as a cause of death in the 19th century in England and Wales has been overstated and conclude: ‘If infections were not the major causes of death a century ago, then surely any major transition is a chimera’. French scholars have vividly argued that in France, the mortality increase hitherto ascribed to cardiovascular diseases may have been an artifact of cause-of-death misclassification of deaths due to ill-defined causes once deaths are properly distributed at least until 1925 (56). They based their contention on their reconstitution of historical series of deaths classified by cause of death on the basis of a constant definition above and beyond the various revisions of the International Classification of Diseases (56). Put differently, MartiInez and Gustavo (44): 543) raised the question ‘Transition … towards what?’. Weisz and Olszynko-Gryn (49): 309) has noted: ‘If Omran essentially ignored chronic disease in most of his work why did he bother including it in his theory? … Omran was a bricoleur who liked connecting everything he knew about a subject’. Not only do some infectious diseases have chronic disease characteristics, but infectious agents and related inflammatory processes are also important in the etiology of a number of chronic diseases and adverse outcomes (57) and preventive programs (58).

Sixth, the epidemiological transition has been criticized for failing to distinguish adequately the risk of dying from any given cause or set of causes from the relative contributions of the various causes of death to overall mortality (59). As patterns of disease and mortality change, there are changes in the relative contribution of different causes to overall mortality that may not reflect changes in actual risk (57). Heuveline et al. (60) have shown that the people in the poorest quintile suffer consistently higher mortality in all three of the major categories of disease (i.e. Group I – Communicable, maternal, perinatal and nutritional conditions; Group II – Non-communicable diseases; Group III – Injuries) used by the World Health Organization than those in the richest quintile, most of the excess mortality being primarily due to the higher risk of communicable diseases. In Africa, both the relative contributions and the actual risk of death from the major cause-of-death categories vary widely across countries, even between countries in the same region, as well as across population groups within a country (61–64). Thus, the epidemiological transition oversimplifies the patterns and relations among risk of mortality, mortality causes, and life expectancy. As substantiated by recent developments in epidemiological methods, the patterns are clearly more complex than simply declining mortality rates from infectious diseases and increasing rates of death from non-infectious diseases (57).

The seventh criticism is that the resurgence of old diseases and emergence of new diseases was not anticipated in the epidemiological transition. In fact, Omran (2) emphasized the fact that whether infectious diseases will ever be extinguished remains a question with a regrettable answer. Indeed, the idea of an ‘epidemiological transition interrupted’ has recently been proposed and discussed (37, 38), disproving Omran's epidemiological transition conceived as a three-stage transition (1). Our cross-examination of evidence from Africa (6) and empirical evidence both historical and contemporary from other studies (26, 37, 38, 43, 48, 51, 59) warns against the operation of a smooth and uninterrupted progression from stage one to stage three and beyond, which was ultimately proposed by Omran (2).

### Why should the health transition be revisited?

Several criticisms of the epidemiological transition just identified apply to the health transition. Moreover, there is no agreed upon definition of health transition or its testable characterization in low-income countries, and the concept can hardly be put into operation without ambiguity (6). Omran (2: 99) stressed that ‘all of the transitions involved in both the dependent and independent variables are subject of epidemiological study and, hence, are encompassed by the epidemiological transition. Epidemiology incorporates the scientific capacity to analyze social, economic, demographic, health care, technological and environmental changes as they relate to health outcomes. Classifying all the changes in these variables under the “health transition” would, however, be confusing. Health is a dependent variable of epidemiology, not vice-versa’. Building on the lessons from the historical experiences of developed countries, much of the decline in mortality in the late 18th century and throughout the 19th century in Western countries preceded the development of modern medicine. For Caldwell, the term ‘health transition’ refers to the driving forces (cultural, social and behavioral change) of improvements in health, with relevance to circumstances of poor economic growth found in many sub-Saharan African countries. It does not address the impact of economic growth and the introduction of modern medicine in such improvements. But, just as one cannot see changes in a society in a stage-wise perspective, so no model can assume that the poor economic growth performance is homogenously invariant in Africa (e.g. Equatorial Guinea is now considered by the World Bank ranking as a high-income country). Chen et al. (65) also challenged the usefulness of the health transition for policy development.

All of these limitations of existing frameworks for investigating demographic and health changes in the epidemiological landscapes of countries around the world, call for a look anew at ways to better understand the mechanisms underlying the exposure to and occurrence of disease, illness, sickness, death and ensuing phenomena.

## Beyond the demographic, epidemiological and health transitions: multilevel eco-epidemiological life course framework for the health, disease and mortality cross-continuum

### Research traditions and general strategy

**Fig. 1:**
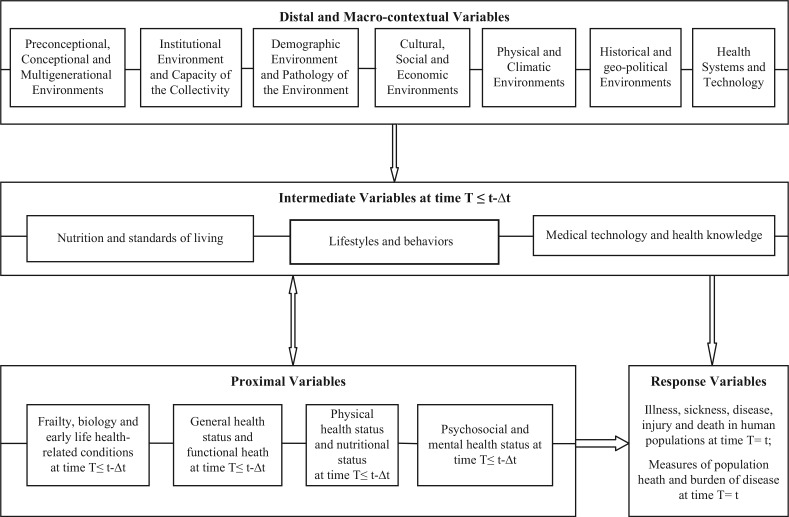
Multilevel eco-epidemiological life course framework for the health, disease and mortality cross-continuum in human populations.

Building from prevailing research traditions in causality, modeling, causal inference and counterfactuals (66–74), health-related analytical frameworks (75–83), and theoretical perspectives just reviewed – demographic transition theory (17), epidemiological transition theory (1, 2) and health transition (18, 19) – we propose a multilevel eco-epidemiological life course framework for the health, disease and mortality cross-continuum for deepening understandings of the demographic and epidemiological changes and multilevel responses of the health care system to them, in the global health context. The framework is depicted in Fig. 1.

This framework links together the mechanisms and processes through which individual micro-level decisions and behaviors, household and family meso-level characteristics, and context-dependent macro-level environments that influence health and longevity aggregate to macro-level demographic and epidemiological profiles, trends and differentials in fertility, mortality, migration, burden of disease and population health patterns in human populations. Such a framework is designed to help construct a precise understanding of these mechanisms for the formulation of research design, planning of interventions and development of health policies which will contribute to the development of healthy societies and promote global health.

Among the many implications of population and health research within a cross-continuum perspective is the potential to improve understanding on how people during their life course stay healthily free of diseases, illness or sickness as well as how long people live over time and space as population ages.

All indications are that the Millennium Development Goals designed by the United Nations to improve the health of the world's poorest will not be met by 2015 (84), most likely not even by the end of this century in most African countries if current trends continue (85).

In a global context of progresses in human longevity and life expectancy, changes in morbidity and disability trends and secular falls in mortality are expected. The idea that populations’ health and disease profiles just as individuals’ health and disease patterns change over time because common diseases have their roots in changes in geography, socioeconomic contexts, social norms, culture, socioeconomic, institutional settings, medical contexts, lifestyle factors, and multifaceted environments of human life, makes sense in preventive medicine (86, 87) and is supported by the new public health (88).

### Cross-continuum of health, illness, sickness, disease, and death

Given the multiple dimensions of health (3) and the need for improving the accurate of measuring, reporting, and interpreting the health state of individuals and populations (86, 87), we propose a framework which articulates the nested structure of influential factors of individual health or population health across the health continuum. This health, disease and mortality cross-continuum framework embodies the continuously varying multilevel eco-epidemiological contexts of exposure to and occurrence of illnesses, sicknesses, diseases, and deaths of individuals nested in local or national populations. The time dimension is factored throughout the framework within the life course perspective (70–82).

The notion of continuum has been incorporated in many dimensions of population health and health care. For instance, the continuum of care is a concept involving an integrated system of care that guides and tracks patient over time through a comprehensive array of health services spanning all levels of intensity of care (77). The definition of the health/disease boundary being inevitably arbitrary (89), there needs to be measures for illness, sickness, disease along a continuum from conception to death in specific contexts, so as to capture the epidemiological profiles of populations. Progress toward preventing premature deaths across the lifespan and alleviating the burden of disease on populations of developing countries and those of sub-Saharan Africa in particular where such burden is greatest for preventable diseases, requires a better grasp on what are the illnesses, sicknesses, diseases and causes of death encountered by individuals in these populations. This is of paramount importance in many developing countries where culture and perceptions still play a major role in reporting and documenting diseases, disabilities and deaths, so that the burden of disease tends to be heavily underestimated by standard measures of health and disability.

The complexities of what constitutes a disease cannot be underestimated and discussing this requires careful distinction among related, but distinct concepts of illness, sickness, disease and death, on the birth-to-death continuum during the life course across space and time (90). The dictionary of epidemiology (91) defines them following Susser (90) who proposed some useful definitions of these concepts: disease is a physiological or psychological dysfunction; illness is a subjective state of the person who feels aware of not being well; sickness is a state of social dysfunction or a role that the individual assumes when ill. Disease progression is often taken to be reflected in the accumulation and severity of symptoms.

From these definitions, illness refers to the subjective sense of feeling unwell; illness does not define a specific pathology, but refers to a person's subjective experience of it, such as discomfort, tiredness, or general malaise (92). Cultural background influences the way a patient reports symptoms. Susser applied the term sickness to refer to socially and culturally held conceptions of health conditions (e.g. the dread of cancer, the stigma of mental illness, misconceptions about HIV/AIDS), which in turn influence how the patient relates to his or her health status and to the health care system. The social perceptions of a disease may affect the extent to which a condition is concealed, may modify the ways a patient perceives his symptoms, and may affect the likelihood of seeking care irrespective of its affordability in the traditional or modern health care systems. Cultural norms and practices likewise complicate the drawing of the boundary between disease and non-disease, since disease implies a focus on pathological processes that may or may not produce symptoms and that result in a patient's illness (92). In the ‘biomedical model’ of disease which focuses on pathological processes and on understanding, diagnosing, and treating the physical and biological aspects of disease, there is a great potential of attribution error and misspecification of disease based on the patient's symptoms. Moreover, there have been changes over time in cut-points for what is a normal value or ‘normal’ when measuring health of people to determine individuals who have a disease and those who do not have a disease. For instance, the cut-points for defining hypertension have changed over time. Prehypertension, a classification for cases where a person's blood pressure is elevated above normal but not to the level considered to be hypertension (high blood pressure) was redefined in 2003 to be blood pressure readings with a systolic pressure from 120 to 139 mm Hg or a diastolic pressure from 80 to 89 mm Hg. This implied revisions of past trends in prevalence of hypertension and increased the incidence and prevalence of hypertension and the associated treatment costs to individuals and the health care system, as most people eventually become hypertensive as they age. It is estimated that more than half of people over age 60 and approximately three-quarters of people over age 70 have hypertension. Since most biological measurements of risk factors of non-communicable diseases produce a continuous range of values (e.g. blood pressure, body mass index), a cut-point on each of these scales has to be chosen to divide the ‘normal’ from the ‘abnormal’ results among a range of findings, which vary from definitely abnormal to definitely normal. Nutritionally, abnormality occurs at both ends of a continuum as being underweight and being overweight are both unhealthy.

The complexity of the relationship between health and illness has inspired important discussions of the nature of medicine and disease (92, 93). The World Health Organization provides a commonly used definition of health: ‘the state of complete physical, mental, and social well-being and not merely the absence of disease’ (94). This definition emphasizes the importance of physical, mental, and social health, suggesting that a breakdown or shift in any of these components may result in poor health but not necessarily disease. It encourages researchers to move away from simply equating health with the absence of clinical disease. This definition also allows health to be culturally defined and experienced by individuals within their cultural systems (95). In other words, illness categories and conditions resulting in poor health may vary between populations. The processes of demographic and epidemiological changes and their responses are also continuous through time, reflexive because a change in one component is eventually altered by the change it has induced in other components of these processes, and behavioral since these process involve human decisions in the pursuit of goals of living a long and healthy life as a fundamental aspect of human development, with varying means and conditions over time and space. The set of illnesses suffered by individuals and populations at a particular time and the process from individual good health to death or from population good health to mortality, are never the same at the next time while continuously connected. Abrupt changes may be more easily perceived than those that take place more slowly over a longer period of time, and these processes are essentially in a continuous state of flux. As a result, the subject has a frightening complexity and intricacies in developed and developing countries alike. In the former, despite massive and often accurate historical and contemporary data on these processes, a number of theoretical and substantive questions remain a matter of debate (51–59). In the latter, the lack of accurate data and studies has been a deterrent to attempting a good picture of the changing contexts of these processes, especially in the African context. The expectation that various developing countries including African countries go through stages and transitions in population structure and health shaped by changes in fertility and mortality by cause of death as they progress toward fuller industrialization as it was documented in now-developed countries of Europe and North America, has not been materialized in many African countries and regions over the last 60 years where infectious diseases remain the predominant causes of illness and death (61, 62, 6).

### Distal, intermediate, proximal, and outcome variables

In the first half of the 20th century, scientific conceptions of causation underwent radical change (66), which has continued to date (67–76). In physics, the development of quantum mechanics led to an acceptance of indeterminism and a rejection of classical concepts of cause and effect; in biology, biostatistics, and the social sciences, there was increasing acknowledgement of the complexity of natural phenomena and the need for a broader concept of causation, which saw causes as multifactorial rather than as a single agent or event. There was also greater acknowledgement of the limitations of scientific measurement and the need to deal with uncertainty about causal mechanisms. Similarly, multilevel and multifactorial causes are involved in the production of health and diseases, and the idea of promoting better health involves tracking the courses of specific diseases. We argue that the continuing rise of chronic infectious diseases and non-communicable diseases usually occurs in a context of uninterrupted interactions between and among communicable and non-communicable diseases across the life course of individuals and communities in developing countries. A multilevel eco-epidemiological model within a life course perspective that captures the health and disease cross-continuum model is better suited for health research in environments typical of those of the vast majority of LMICs.

This perspective recognizes multiple and interrelated levels of causation, offering the possibility for models that are more integrated rather than fragmented. The implication of this framework for research and practice designed for health improvements has three pillars. The first is the life course perspective which requires thinking in terms of changes in causal pathways across the life span when considering health and disease patterns in human populations. Second, the causal models on which to rely must allow for multiple levels of determinants acting in complex and interrelated ways, often synergistically or with feedback loops or reciprocal lines of causality, given the interactions among diseases. Furthermore, we consider that higher-level determinants may have emergent properties above and beyond the aggregate of their constituent parts. Finally, when considering the multiple levels of this life course eco-epidemiological model, we rely on the understanding that disease occurs in individuals, but interventions can occur at any level, including individual-level, family-level, community-level, resulting in healthy people in healthy communities.

This perspective sees the whole range of determinants as integral to individual, family, community and national health and well-being. Such life course eco-epidemiological model, akin to the modern ecological model of public health practice, stresses the multiple dimensions that constitute our lives, relationships, and environments, hence contributing to wellness or disease, disability and death along the health, disease and mortality cross-continuum in human populations.

Distal and macro-contextual variables (multilevel 4). A distal factor is a factor distant in time to the event-outcome. Distal-level variables include stable dispositional variables and environments that predate the intermediate and the (immediate) proximal contexts. It has been shown that environmental processes influencing the propensity to disease in adulthood operate during the preconceptional, conceptional/fetal, infant phases of life, and throughout the life course. Distal variables include the various environments which protect from or expose to various health-related outcomes: 1) preconceptional, conceptional, and multigenerational environments (80, 81, 96–99); 2) institutional environment and capacity of the collectivity (100, 101); 3) demographic environment and pathology of the environment (86, 87, 102); 4) cultural, social, and economic environments (86, 87); 5) physical and climatic environments (102); 6) historical and geopolitical environments (6); and 7) health systems and technology (103, 104). The idea that social conditions are root causes of disease and health of populations originated from McKeown (105, 106) over half a century ago, and the World Health Organization followed suit only recently by creating a Commission on Social Determinants of Health (107). McKeown's thesis states that the enormous increase in population and dramatic improvements in health that humans have experienced over the past two centuries owe more to changes in broad economic and social conditions than to specific medical advances or public health initiatives.

Intermediate variables (multilevel 3). An intermediate variable in a causal pathway is a variable that causes variation in the response variable and is itself caused to vary by the distal variables (108). Intermediate variables include: 1) nutrition and standards of living (109); 2) lifestyle and behaviors (102); and 3) medical technology and health knowledge (102–104).

The proximal-level variables (multilevel 2). They include the immediate settings, contexts, or conditions prevailing prior to the occurrence of the outcome of interest. A proximal factor is a factor close in time to the event or onset of the behavior of interest as outcome variable. Proximate variables include: 1) frailty, biology, and early life health-related conditions (104, 110, 111); 2) general health status and functional health (102, 111); 3) physical health status and nutritional status (102, 109); and 4) psychosocial and mental health status (112).

The response variables (multilevel 1). They may include both individual-based measures (illness, sickness, disease, injury, and death) as well as measures of population health and burden of disease.

The intermediate variables are determined by the groups of distal factors, including the demographic environment (e.g. population density, urbanization, rural–urban migration, population composition, population structure by age and sex); the capacity of the collectivity (e.g. agricultural and food security); the cultural and social environments (e.g. ethnicity, means of communication such as telephone and cellular phones, norms and practices that have bearing on health and survival in the life course); the economic environment (e.g. per capita gross domestic product, consumer price index, national macroeconomic and microeconomic foundations, fiscal policies); the physical and public health environments (e.g. improved measures of hygiene, sanitation, water access, preservation of the environment); and the health system and technology (e.g. health services, infrastructure and equipment, skilled personnel and quality of care).

Changes in intermediate variables (nutrition and standards of living, lifestyles and behaviors, medical technology and health knowledge including access to and quality of health services) are mostly responsible for the improvements in survival and longevity in contemporary societies just as they accounted for most of the reductions of mortality and increases in life expectancy in developed countries. The technological advances in prevention and treatment of communicable, maternal, perinatal, and nutritional conditions responsible for the majority of deaths have significantly impacted the role of these factors in mortality declines and health improvements over time.

### Nonlinearity in the epidemiology of complex health and disease processes

Nonlinearity in the epidemiology of complex health and disease processes is well documented (113). Around the world, it is not infrequent to see during a visit at a health clinic that many patients will come with multiple conditions, both infectious and chronic or communicable and non-communicable. So, the concept of the cross-continuum of health, disease, and mortality proposed here is both conceptually and empirically appealing.

This cross-continuum is chronologically spanning the period from the beginning to the end (for human life) or for a societal health burden (since there is always in a society some degree of health problems, being communicable, non-communicable, or injuries) and with probability varying from 0 to 1. Therefore, it is possible to develop standardized indicators along the health, disease, and mortality cross-continuum for each society so that they can allow international comparisons. Indeed, various health states are the outcomes ranging from complete absence of ill-health to demise as part of the continuum of health burden (from 0 to 1 on the probability scale) and result from multilevel determinants and consequences at the micro, meso, and macro levels of each society.

The discontinuity in health states is an empirical matter rather than a conceptual or theoretical issue. In particular, any measure of health state is at least in part a reflection of social, cultural, bio-behavioral, economic, physical, and medical influences within the broad context of risk, protective and resilience factors forming a continuum of multilevel states and renewal processes in the nonlinear and dynamic epidemiology of population change.

## Data needs to meet the challenges of, and health sector responses to, the cross-continuum of health, disease, and mortality

The proposed framework provides a methodological foundation for designing data collection and conducting analysis of experiments and quasi-experiments as well as probabilistic samples for capturing the changing demographic and epidemiological profiles of national populations and responses from the health sector to these changes. This framework is multidisciplinary and cut across panoply of theories in fields including economics, environment, climatology, physical and environment sciences, social and biomedical sciences; it is trans-theoretical. It also embraces the life course perspective given the fact that for most chronic conditions and diseases, their development until they reached the disease state proceeds over years or decades. It is also increasingly established the influences of early life conditions on later life health and survival, along this cross-continuum framework.

The variability in the availability and quality of data on mortality statistics, health and aging and cause of death, and the use of different frameworks for conducting research have invariably produced inconsistent and even contradictory perspectives on change and thus on the implications of health assessments for health policy.

There are growing numbers of datasets spanning multiple time periods in LMICs, and researchers are beginning to face the challenges of how to incorporate this longitudinal dimension into their studies in order to capture any significant change over time. Implementing simultaneously comparative and longitudinal models to such repeated cross-sectional data should provide insightful understanding on the spatial effects that operate at different levels to influence health outcomes and social change. By using the proposed framework for assessing demographic and epidemiological changes, it is possible to collect, analyze and use comparable and high quality data on communicable and non-communicable diseases, accidents and other disabilities. Such endeavor will have implications for the demand for health care, for the types of new health care delivery systems and human resources required for disease prevention and health promotion, and for their costs. Available evidence indicates that Africa in general and sub-Saharan Africa in particular constitutes the poorest and least developed regions in the world with the heaviest burden of disease irrespective of indicators used.

Recent patterns and trends of urban growth in developing countries indicate that over half of the world's total population now lives in urban settings. In many developing countries, the contribution of natural growth to the urban to rural growth differential is higher and is seriously outstripping the capacity of most cities to provide adequate services for their citizens and the deteriorating living conditions of the urban poor. The challenges of achieving sustainable urban development will be particularly formidable in Africa. In the coming decades, the world's rapid urbanization will be one of the greatest challenges to ensuring human welfare and a viable global environment. According to current estimates, cities occupy 4% or less of the world's terrestrial surface, yet they are home to almost half the global population, consume close to three-quarters of the world's natural resources, and generate three-quarters of its pollution and wastes (114). New data collection efforts within the framework proposed here should be designed so as to make progress at understanding the role of migration in population health patterns. Such progress which has been hampered by serious methodological issues, paramount among which are: the lagged effects of migration on health, disease, and mortality patterns and differentials between rural and urban settings; the interactions between demographic and epidemiological responses, including interactions of migration patterns with concurrent changes in the demographic and epidemiological profiles of populations; and the effects of political shocks and social crises which are historically adamant in most African countries and hamper health development by perpetuating vulnerabilities in populations and insubstantialities of institutions.

With the availability of most standard software suites (e.g. SPSS, SAS, and R), estimation of complex models using this framework is now possible in most research environments globally.

## Conclusions

The epidemiological transition has been useful in laying out an overarching perspective on changing demographic and diseases patterns in developed countries at least through the 1950s. Its various criticisms suggest that it is relevant as a way of describing and understanding to some extent the relation among disease and mortality patterns in the course of population change in Western societies until the 1950s, rather than as a universal description or prediction regarding population health patterns enlightening to the formulation of health policies in contemporary societies or in developing countries. The historical and contemporary demography and epidemiology of these countries are quite distinct from historical experiences of the Western societies. Moreover, they are faced with enormous and unprecedented disease burdens in tandem with ill-equipped, poorly funded and often dysfunctional health care system in social contexts where the family largely remains the sole source of social security and health insurance for the majority of people faced with disease and risks of premature death.

In his Inaugural Address on the 20th of January 1949, US President Harry S. Truman noted: ‘… More than half the people of the world are living in conditions approaching misery. Their food is inadequate. They are victims of disease. Their economic life is primitive and stagnant. Their poverty is a handicap and a threat both to them and to more prosperous areas. For the first time in history, humanity possesses the knowledge and skill to relieve the suffering of these people’. Over 60 years since this Address, few people will question the maxim that humanity has failed the vast majority of populations in Asia, Latin America and Africa. Why are poverty, malnutrition, disease and suffering, which are all avoidable given the proven interventions and measures which have created healthy environments for living, still rampant in so many societies of the developing world? The 11 articles in this Special Issue coupled with the editorial, have clarified important aspects of the answer to this question posed to the conscience of humanity and there are specific proposals for action made in each article: equipped with this new knowledge and the proposed framework in global health, the words of President Truman should not continue to largely fall on deaf ears of the international community.
